# Primary plant nutrients modulate the reactive oxygen species metabolism and mitigate the impact of cold stress in overseeded perennial ryegrass

**DOI:** 10.3389/fpls.2023.1149832

**Published:** 2023-03-31

**Authors:** Muhammad Ihtisham, Mirza Hasanuzzaman, Ahmed H. El-Sappah, Fawad Zaman, Nawab Khan, Ali Raza, Mohammad Sarraf, Shamshad Khan, Manzar Abbas, Muhammad Jawad Hassan, Jia Li, Xianming Zhao, Xin Zhao

**Affiliations:** ^1^ College of Agriculture, Forestry, and Food Engineering, Yibin University, Yibin, Sichuan, China; ^2^ Key Laboratory of Horticultural Plant Biology, College of Horticulture and Forestry Science, Huazhong Agricultural University, Wuhan, China; ^3^ Department of Agronomy, Faculty of Agriculture, Sher-e-Bangla Agricultural University, Dhaka, Bangladesh; ^4^ Department of Genetics, Faculty of Agriculture, Zagazig University, Zagazig, Egypt; ^5^ College of Management, Sichuan Agricultural University, Chengdu, Sichuan, China; ^6^ Chengdu Institute of Biology, University of Chinese Academy of Sciences, Beijing, China; ^7^ Department of Horticultural Sciences, Faculty of Agriculture, Shahid Chamran University of Ahvaz, Ahvaz, Iran; ^8^ School of Geography and Resources Science, Neijiang Normal University, Neijiang, China; ^9^ College of Grassland Science and Technology, Sichuan Agricultural University, Chengdu, Sichuan, China

**Keywords:** abiotic stress, antioxidant defense system, Cynodon dactylon, Lolium perene, NPK fertilization, oxidative stress

## Abstract

Overseeded perennial ryegrass (*Lolium perenne* L.) turf on dormant bermudagrass (*Cynodon dactylon* Pers. L) in transitional climatic zones (TCZ) experience a severe reduction in its growth due to cold stress. Primary plant nutrients play an important role in the cold stress tolerance of plants. To better understand the cold stress tolerance of overseeded perennial ryegrass under TCZ, a three-factor and five-level central composite rotatable design (CCRD) with a regression model was used to study the interactive effects of nitrogen (N), phosphorus (P), and potassium (K) fertilization on lipid peroxidation, electrolyte leakage, reactive oxygen species (ROS) production, and their detoxification by the photosynthetic pigments, enzymatic and non-enzymatic antioxidants. The study demonstrated substantial effects of N, P, and K fertilization on ROS production and their detoxification through enzymatic and non-enzymatic pathways in overseeded perennial ryegrass under cold stress. Our results demonstrated that the cold stress significantly enhanced malondialdehyde, electrolyte leakage, and hydrogen peroxide contents, while simultaneously decreasing ROS-scavenging enzymes, antioxidants, and photosynthetic pigments in overseeded perennial ryegrass. However, N, P, and K application mitigated cold stress-provoked adversities by enhancing soluble protein, superoxide dismutase, peroxide dismutase, catalase, and proline contents as compared to the control conditions. Moreover, N, P, and, K application enhanced chlorophyll *a*, chlorophyll *b*, total chlorophyll, and carotenoids in overseeded perennial ryegrass under cold stress as compared to the control treatments. Collectively, this 2−years study indicated that N, P, and K fertilization mitigated cold stress by activating enzymatic and non-enzymatic antioxidants defense systems, thereby concluding that efficient nutrient management is the key to enhanced cold stress tolerance of overseeded perennial ryegrass in a transitional climate. These findings revealed that turfgrass management will not only rely on breeding new varieties but also on the development of nutrient management strategies for coping cold stress.

## 1 Introduction

Bermudagrass (*Cynodon dactylon* (L) Pers.) turf is an extensively used warm season turfgrass in sports fields, golf courses, parks, and greenbelts throughout the world (Ihtisham et al., 2020). The optimal temperature for its best growth and performance ranges from 26°C–35°C (Fan et al., 2014). Low temperature severely restricts its growth and performance (Huang et al., 2019) and therefore low temperature is considered a key abiotic factor that confines the use of bermudagrass (Ihtisham et al., 2018). A transitional climatic zone (TCZ) refers to the transitional zone between arid and humid regions that is sensitive to climatic fluctuations (Barker et al., 2019). In China, TCZ is located in marginal areas of the East Asian monsoon zone where the ecosystem has become unstable due to combined effects of climatic change and human activities (Yin et al., 2021; Yin et al., 2022). TCZ possesses both severe cold and hot temperatures in their respective seasons (Christians et al., 2016). As a result, it’s difficult for warm-season turfgrass to perform well in winter, and cool-season turfgrass to perform better in summer (Ihtisham et al., 2018). To overcome the winter dormancy of warm-season turfgrasses, overseeding practice with a cool-season turfgrass is commonly practiced (Shi et al., 2014). Perennial ryegrass (*Lolium perenne* L.) is an important cool-season turfgrass that is frequently overseeded on warm-season grasses for the temporary re-establishment of turfgrass cover, color, playability, functionality, and aesthetic value (Xie et al., 2020). The overseeding practice comprises sowing cool-season grass seeds over established dormant warm-season turfgrass (Ozkan and Kir, 2021). Despite a cool-season turfgrass, perennial ryegrass is considered susceptible to utmost cold stress (Hoffman et al., 2010).

Future climatic change predictions are increased summer temperature, higher rainfall, longer growing seasons, and more irregular winter climates (Ostrem et al., 2018; IPCC, 2021). The anticipation and frequency of utmost temperatures (cold and hot) will provoke instability, and the winter short photoperiods with extremely low temperatures in transitional zones will drastically affect cool-season turfgrasses (Uleberg et al., 2014; IPCC, 2021). Cold stress is a major environmental factor that limits crop productivity and poses a serious threat to agricultural sustainability (El-Sappah and Rather, 2022). Cold stress negatively affects cellular components and metabolism, and imposes stresses of variable severity that depend on the intensity and duration of the stress. Cold stress leads to chlorosis, necrosis, changes in cytoplasm viscosity, membrane damage, and changes in enzyme activities leading to plant death (Atayee and Noori, 2020). Moreover, cold stress disrupts the integrity of intracellular organelles, leading to the loss of compartmentalization, reduction and impairment of photosynthesis, protein assembly, and general metabolic processes (Bera et al., 2022). The effects of cold stress cause great challenges to the utilization of overseeded cool-season turfgrasses globally (Xie et al., 2020). Plants under optimum growing conditions usually maintain reactive oxygen species (ROS) through stable production and scavenging mechanisms. However, during stress conditions, this balance is perturbed (Dahro et al., 2016; Hussain et al., 2016; Sarraf et al., 2020; El-Sappah et al., 2021a). The increased accumulation of ROS such as hydrogen peroxide (H_2_O_2_) under stress causes membrane lipid peroxidation (MDA) and electrolyte leakage (EL), which are effective indicators of oxidative stress (Hu et al., 2018; El-Sappah et al., 2021b). Numerous approaches are being used to acclimate or mitigate the harmful effects of cold stress, such as the application of plant nutrients (macro and micro), plant growth regulators (jasmonic acid, abscisic acid, salicylic acid, brassinosteroids, and gibberellin), the unitization of genetics tools, and plant breeding (Huang et al., 2018; Atayee and Noori, 2020; Faiq and Noori, 2021).

Plant primary nutrients play crucial functions in the growth and development of plants and in improving cold stress tolerance as they are intimately involved in the plant cell organization and metabolic functions. The mineral nutrients are either structural/functional constituents of enzymes or act as activators or regulators of various enzyme activities. The macronutrients such as nitrogen (N) and phosphorus (P) form structural constituents of building block materials, and some of them like potassium (K), calcium (Ca), and magnesium (Mg) are regulators or activators of enzymes (Zhang et al., 2017). Deficiencies of these nutrients impact various physiological or metabolic activities including increased production of (ROS) that cause oxidative stress in plants (Tewari et al., 2021). Mineral nutrients such as N, P, and K play a critical role in temperature stress tolerance and adequate application is vital for the integrity of plant structure and key physiological processes such as N is a structural part of chlorophyll needed for photosynthesis, P is needed for energy production and storage, is a structural part of nucleic acids, and K is needed for osmotic regulation and activation of enzymes (Waraich et al., 2011; Waraich et al., 2012).

Previous studies of cold stress and nutrient deprivations also showed increased ROS accumulation (Hussain et al., 2016; Huang et al., 2017). Higher plants retain extremely efficient antioxidant defense systems to scavenge excessive ROS against oxidative stress (El-Sappah et al., 2023). Stress-induced damages are alleviated by several enzymatic (superoxide dismutase, SOD; peroxide dismutase, POD; catalase, CAT), and non-enzymatic pathways (Dahro et al., 2016; Hussain et al., 2016). Turfgrass performance is controlled by optimum N, P, and K supply while their deficiency is a key limiting factor. N is a vital element of proteins, amino groups, Rubisco, and chlorophyll that regulates enzymes essential for stress tolerance (Tantray et al., 2020). P is the main part of molecules that are crucial at cellular levels, for example, it is in ADP, ATP, phospholipids, and nucleic acids. Similarly, K is an inevitable part of cellular homeostasis, enzyme catalysis, and osmotic adjustments (Johnson et al., 2022; Zaman et al., 2022).

We conducted a series of experiments on overseeded perennial ryegrass over dormant bermudagrass under a transitional climate. The first experiment was published in 2018 where we studied the morphological, phenotypic, qualitative, and gas-exchange parameters, and optimized the rates of N, P, and K fertilization (N: 30, P: 24, K: 9 and N: 30, P: 27, K: 6 g m^-2^ respectively, during two years) for integrated turf performance (Ihtisham et al., 2018). Although many studies documented the role of N, P, K fertilization in alleviating oxidative stress caused by severe cold. Till now, there is very limited research on the combined effects of N, P, and K fertilization on oxidative stress of overseeded perennial ryegrass under cold stress in transitional climates and with the use of central composite design. The ultimate goals of this study were to examine that primary plant nutrients (N, P, and K) play an important role in the cold stress tolerance of overseeded perennial ryegrass under TCZ. Therefore, this study was performed to assess the interactive effects of N, P, and K fertilization on lipid peroxidation, electrolyte leakage, ROS production, and their detoxification by the photosynthetic pigments, enzymatic and non-enzymatic antioxidants defense of overseeded perennial ryegrass under cold stress conditions in the transitional climate.

## 2 Materials and methods

### 2.1 Study site and experimental operations

**Figure 1:**
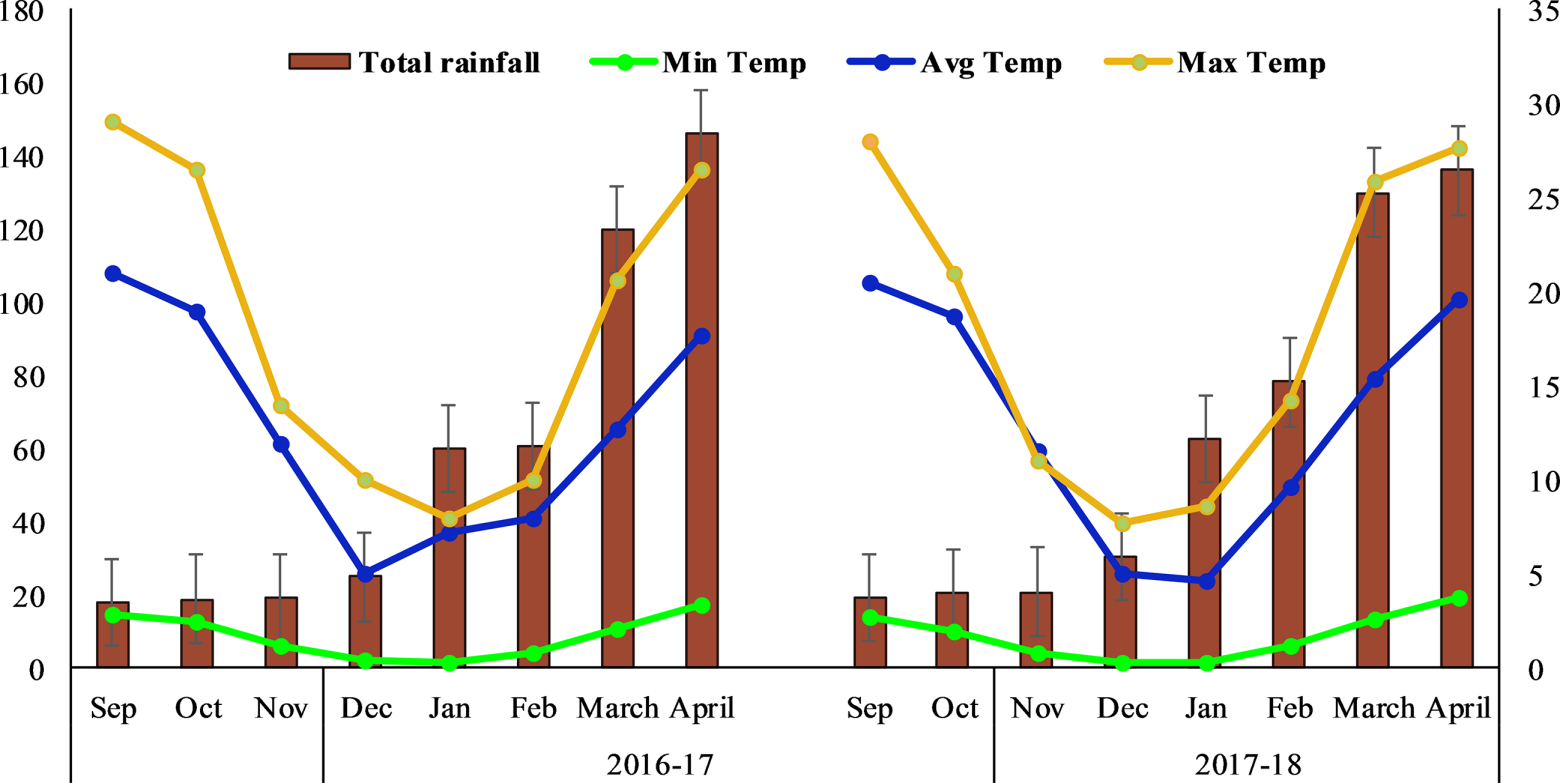
Maximum, minimum, and average temperatures and mean annual rainfall in the study location.

The two years study was conducted from autumn 2016 to summer 2018 at Huazhong Agriculture University, Wuhan-China (114^°^E, 30^°^N). Weather data for two years were attained from the university weather station (**Figure 1**). The area has a transitional climatic zone with an average yearly precipitation of 1150–1450 mm and a subtropical monsoon climate. During the two years, the recorded average temperature was 4 – 21°C and 4.5 – 21.5°C, respectively. Moderate snowfall was recorded in December and January during both years. The soil at the experimental site has a loess texture and prior to experimentation, soil samples at the depth of 15 cm were randomly collected for physiochemical properties (**Supplementary Figure S1**).

Field treatments were arranged in 24 plots of 2m length and 1.5m width. Hybrid bermudagrass [*C. dactylon (L.)* Pers.] “Tifway419” was established from plugs at the start of May each year. A starter dose of N, P, and K fertilization of 10 g m^−2^ each was applied at the initiation of new growth to ensure high-quality turf for overseeding. In the autumn of each year, overseeding experimentations were performed during the second week of October. A verticut of 1 cm height was applied to bermudagrass and perennial ryegrass (*Lolium perenne L.*) seeds were overseeded at 25 g m^−2^ density, followed by sprinkle irrigation. The plots were fertilized after full germination (fourth week) with combinations of N as Urea (N 46.4%), P as single superphosphate (P_2_O_5_ 16–18%), and K_2_O as potassium sulfate (K 50%) (**Table 1**). Overseeded perennial ryegrass cover reached <80% after four weeks of fertilization in the treated plots.

**Table 1:** Experimental variables, codes, and coded levels in central composite rotatable design (CCRD).

Experiment variables	Coded symbols	Coded and actual variable levels				
		–1.682	–1	0	1	1.682
Nitrogen (N)	X_1_	0	6.082	15	23.918	30
Phosphorus (P)	X_2_	0	6.082	15	23.918	30
Potassium (K)	X_3_	0	6.082	15	23.918	30

X_1_, X_2_, X_,3_, represent coded symbols for N, P, K. –1.682 to 1.682 represent coded and 0, 6.082, 15, 23.918, 30 indicate actual levels of N, P, and K.

### 2.2 Sampling and quantification of biochemical analysis

Following the snowfall in December and January during both years when the snow melted and the average daily minimum temperature was 0 – -5°C, tissue samples (leaves) of fresh overseeded perennial ryegrass were detached and immediately stored at −80°C for biochemical analysis. Soluble protein, SOD, POD, CAT, MDA, and H_2_O_2_ were determined according to instructions provided by assay kits manufacturer (Nanjing Jiancheng Bioengineering Institute, China). Proline was determined according to the acid-ninhydrin method, EL by conductivity meter, and chlorophyll *a*, *b*, total chlorophyll, and carotenoids by spectrophotometer.

### 2.3 Crude enzyme extraction and the determination of soluble protein, enzymatic antioxidants, and proline content

For the determination of soluble protein, SOD, POD, CAT, and proline, the crude enzyme was extracted by grounding 0.5 g of tissue samples in an automatic ice-cooled mortar and pestle. The tissue powder was immersed in 4 mL phosphate buffer (150 mM, PH, 7.0), precooled at 4°C and the homogenate was transferred into 10 mL plastic tubes for centrifugation at 10,000 rpm for 15 minutes at 4°C. The resulting supernatant was collected and stored at 4°C.

Soluble protein, SOD, POD, and CAT activities were measured spectrophotometrically and expressed as units mg^−1^ protein, according to the manufacturer’s instructions. These kits were purchased from the Nanjing Jiancheng Bioengineering Institute, China. The kits used were (A045-2), (A001), (A084-3), and (A007-1) for soluble protein, SOD, POD, and CAT, respectively, and expressed as enzyme units per mg of protein [U mg^–1^(protein)]. Proline content in fresh overseeded ryegrass leaves was appraised spectrophotometrically using the acid-ninhydrin method according to the protocol of Bates et al (1973). By using 4 mL toluene, the reaction mixture was extracted and read at 520 nm. The proline concentration was expressed as micrograms per gram fresh weight (μg g^-1^ FW) using a standard curve developed with proline.

### 2.4 Determination of EL, MDA, and H_2_O_2_

EL in the overseeded ryegrass leaves was determined according to the known method of Bates et al (1973). Initial conductivity (C_1_) and final conductivity (C_2_) were measured by a conductivity meter (model: DSS-307, SPSIC, China) and EL was calculated using the formula:


(1) $$EL\left(\%\right)=\left(\frac{C_{1}}{C_{2}}\right)\times 100$$

MDA and H_2_O_2_ were also determined using the appropriate assay kits from the same company. The manufacturer’s instructions were followed, and MDA and H_2_O_2_ were expressed as (nmol mg^-1^ protein).

### 2.5 Quantification of photosynthetic pigments

The photosynthetic pigments viz. chlorophyll *a* (Chl *a*), chlorophyll *b* (Chl *b*), total chlorophyll (T.Chl), and carotenoids (Car) were extracted with 80% acetone by the previously used known methods (Marcum, 1998).

### 2.6 Experimental design and statistical analysis

The experiments were arranged using a central composite rotatable design (CCRD) for the assessment of interactive effects of N, P, and K fertilization on MDA, EL, electrolyte leakage, and ROS production and their detoxification by photosynthetic pigments, enzymatic, and non-enzymatic antioxidants defense system of overseeded perennial ryegrass under cold stress.

In this design, a total of five levels (0, 6.082, 15, 23.918, and 30 g m^-2^) for each of the three factors (N, P, and K) were used. The CCRD consisted of 2^3^ factorial runs with 8–factorial points, 6–axial points, and 9–replications at the center point. There is also a control treatment (N_0_P_0_K_0_), comprising 24 experimental treatments as determined by equation (2).


(2) $$\text{A }=\;2^{\text{b}}+\;2\text{b }+{\text{ d}}_{0}$$

where A=no. of experimental runs, b= no. of factors, and d_0_=no. of center point replications.

Theoretically, replication of the central treatment in central composite designs is very important as it provides an independent estimate of the experimental error and it should be repeated more than five times. Therefore, in our study, we have repeated the center point treatment nine times to precisely determine value, while the replication of other treatments is not needed (Montgomery, 2001). The total experimental treatments were calculated as (Treatments= 2^3^ + 2×3+9+1 = 24).

Treatments using various regimes of N, P, and K doses were coded (**Table 1**) using equation (3):


(3) $$\text{Z}i={\text{z}}_{i}-{\text{z}}_{i}0/\Delta {\text{z}}_{i}$$

Where Zi= ith variable coded value, z_i_= ith variable real value, z_i_^0^= ith variable real value at the center point, and Δz_i_= step change value.

The experimental runs, coded values, and applied doses are shown in (**Supplementary Table S1**).

The interactive effects of different treatment combinations on biochemical parameters were analyzed with a second-order polynomial equation (4).


(4) $$“Y=a_{0}+a_{1}x_{1}+a_{2}x_{2}+a_{3}x_{3}+a_{4}x_{1}x_{2}+a_{5}x_{1}x_{3}+a_{6}x_{2}x_{3}+a_{7}x_{1}x_{1}+a_{8}x_{2}x_{2}+a_{9}x_{3}x_{3}”$$

Here, Y= response variable; a_0_= constant-coefficient; a_1_, a_2_, a_3_= linear coefficients; a_4_, a_5_, a_6_= interaction coefficients; a_7_, a_8_, a_9_= the quadratic coefficients. x_1_, x_2_, x_3=_ N, P, and K coded values.

The interactive effects of N × P, N × K, and P × K for each parameter were obtained by transforming the data according to equations (5) and (6).


(5) $$X=\frac{x\;-\;x_{min}}{x_{\max-\;x_{min}}}$$

(6) $$X=1\;-\frac{x\;-\;x_{min}}{x_{\max-\;x_{min}}}$$

Where *X* represents the transformed value and *x* represents the observed value of each treatment, and x_max_ and x_min_ represent maximum and minimum values, respectively.

Equation (5) was used for those indices that were positively correlated with N, P, and K application. However, Equation (6) was used for the indices that were negatively correlated with N, P, and K application, as suggested by previous studies (Li et al., 2019; Roy et al., 2021).

For each variable, an independent linear regression was carried out using the above equation. The SPSS (ver. 16) was utilized to find out the constant, regression coefficient of linear, quadratic, and interaction terms. The significance level of individual and interactive effects of independent variables (N, P, and K) was judged by using an *F-value* at *P <* 0.05. The coefficient of determination (R^2^) was used to determine the adequacy of regression equations. For statistical and regression analysis, Microsoft Excel 2018 and SPSS were used, while for correlation analysis and figures, the Origin 2021 software (OriginLab Co., Northampton, MA, USA) was applied.

## 3 Results

We investigated a variety of stress-related biochemical parameters during the two years (2016-2017) and (2017-2018) for overseeded perennial ryegrass under different fertilization treatment combinations (**Tables 2**–**4**) and their regression coefficients with coefficients of determination (R^2^) for each parameter (**Tables 5**–**7**). Pronounced variations in these parameters were detected under cold stress and fertilization.

**Table 2:** Cold stress-induced MDA, EL, and H_2_O_2_ contents of overseeded perennial ryegrass leave under different treatment combinations of N, P, and K fertilization in 2016-17 and 2017-18.

Treatments	MDA (nmol mg^-1^ protein)		EL (%)		H_2_O_2_ (nmol mg^-1^ protein)	
	2016-17	2017-18	2016-17	2017-18	2016-17	2017-18
N_23.918_ P_23.918_ K_23.918_	42.59	44.60	82.70	85.45	1.57	1.63
N_23.918_ P_23.918_ K_6.082_	36.80	36.60	84.31	85.76	1.51	1.53
N_23.918_ P_6.082_ K_23.918_	41.03	39.07	79.73	85.62	1.51	1.56
N_23.918_ P_6.082_ K_6.082_	43.69	46.81	83.33	87.58	1.59	1.68
N_6.082_ P_23.918_ K_23.918_	59.98	59.88	87.79	90.36	1.85	1.88
N_6.082_ P_23.918_ K_6.082_	45.55	54.96	89.58	87.40	1.69	1.78
N_6.082_ P_6.082_ K_23.918_	50.78	50.93	89.01	90.62	1.75	1.77
N_6.082_ P_6.082_ K_6.082_	61.09	57.22	86.16	90.35	1.84	1.84
N_30_ P_15_ K_15_	39.87	46.06	86.14	88.15	1.58	1.68
N_0_ P_15_ K_15_	64.36	65.92	91.14	92.33	1.94	1.98
N_15_ P_30_ K_15_	26.30	42.64	85.70	87.80	1.40	1.63
N_15_ P_0_ K_15_	47.41	54.35	85.66	85.19	1.66	1.74
N_15_ P_15_ K_30_	48.37	49.42	85.87	88.47	1.68	1.72
N_15_ P_15_ K_0_	44.75	54.70	85.69	86.30	1.63	1.76
N_15_ P_15_ K_15_	30.27	30.67	85.85	88.81	1.45	1.49
N_15_ P_15_ K_15_	29.77	37.06	87.27	82.90	1.46	1.50
N_15_ P_15_ K_15_	21.22	45.65	86.88	88.87	1.35	1.68
N_15_ P_15_ K_15_	31.58	36.65	83.38	87.59	1.44	1.55
N_15_ P_15_ K_15_	29.87	36.85	80.07	89.89	1.37	1.58
N_15_ P_15_ K_15_	45.35	31.73	86.38	88.99	1.65	1.51
N_15_ P_15_ K_15_	47.21	37.76	87.37	90.17	1.68	1.60
N_15_ P_15_ K_15_	25.74	42.74	86.40	86.75	1.40	1.62
N_15_ P_15_ K_15_	24.34	30.07	84.29	89.45	1.36	1.49
N_0_ P_0_ K_0_	67.22	70.14	92.15	93.75	1.99	2.05

**Table 3:** Soluble protein, enzymatic antioxidants activities, and proline contents of overseeded perennial ryegrass leaves under cold stress and different treatment combinations of N, P, and K fertilization in 2016-17 and 2017-18.

Treatments	Soluble Protein (g L^-1^)		SOD (U mg^-1^ protein)		POD (U mg^-1^ protein)		CAT (U mg^-1^ protein)		Proline (μg g^-1^ FW)	
	2016-17	2017-18	2016-17	2017-18	2016-17	2017-18	2016-17	2017-18	2016-17	2017-18
N_23.918_ P_23.918_ K_23.918_	2.09	1.98	216.96	214.07	110.98	101.03	12.97	12.43	68.19	73.96
N_23.918_ P_23.918_ K_6.082_	2.22	2.14	225.41	206.17	117.30	104.25	10.07	9.47	72.79	79.06
N_23.918_ P_6.082_ K_23.918_	1.49	1.64	149.90	193.89	78.99	82.93	20.98	21.12	63.86	65.60
N_23.918_ P_6.082_ K_6.082_	2.06	1.67	212.50	182.92	108.45	89.06	17.07	20.55	63.99	66.74
N_6.082_ P_23.918_ K_23.918_	0.91	0.94	80.43	85.71	47.49	47.39	5.74	1.06	57.68	49.40
N_6.082_ P_23.918_ K_6.082_	1.46	1.15	120.89	117.93	74.68	57.66	2.60	7.92	55.64	52.55
N_6.082_ P_6.082_ K_23.918_	0.99	0.96	86.36	92.04	51.23	47.84	3.83	7.40	54.70	55.57
N_6.082_ P_6.082_ K_6.082_	1.51	1.09	134.93	109.96	68.62	53.03	3.41	2.68	52.42	47.08
N_30_ P_15_ K_15_	1.62	1.56	177.16	170.96	71.64	78.37	19.51	17.36	69.60	55.10
N_0_ P_15_ K_15_	0.78	0.89	70.74	87.501	37.40	42.39	2.71	1.22	45.23	37.45
N_15_ P_30_ K_15_	1.30	1.72	136.23	193.53	59.88	83.62	9.95	9.56	48.96	48.89
N_15_ P_0_ K_15_	0.94	1.15	98.88	114.8	42.07	57.28	19.40	12.33	46.61	52.89
N_15_ P_15_ K_30_	1.30	1.12	134.65	116.15	64.73	54.86	19.03	19.55	65.91	56.61
N_15_ P_15_ K_0_	1.12	1.43	114.71	159.48	57.16	70.09	16.85	16.35	75.87	64.56
N_15_ P_15_ K_15_	1.02	1.02	91.22	108.46	52.24	48.79	18.66	20.97	59.70	48.86
N_15_ P_15_ K_15_	1.09	1.77	107.26	195.97	56.13	86.85	20.13	15.09	68.69	49.36
N_15_ P_15_ K_15_	1.23	1.17	112.46	122.88	61.73	57.80	21.24	17.29	75.50	60.03
N_15_ P_15_ K_15_	0.96	1.80	75.43	194.35	48.86	84.93	21.92	8.74	55.60	45.77
N_15_ P_15_ K_15_	1.30	1.15	136.70	123.15	65.31	53.65	17.81	13.47	50.87	45.50
N_15_ P_15_ K_15_	1.20	1.17	119.66	130.49	59.28	57.41	19.55	10.78	46.01	38.79
N_15_ P_15_ K_15_	1.90	1.54	182.28	168.63	92.60	79.11	14.67	15.21	54.09	42.11
N_15_ P_15_ K_15_	1.36	1.67	148.58	181.6	64.91	81.00	21.39	11.97	70.27	51.24
N_15_ P_15_ K_15_	1.20	1.69	129.55	181.35	58.42	105.79	17.37	12.69	75.03	38.79
N_0_ P_0_ K_0_	0.52	0.39	48.55	38.87	26.67	18.85	1.73	1.62	42.95	34.66

**Table 4:** Chloroplastic pigments of overseeded perennial ryegrass leaves under cold stress and different treatment combinations of N, P, and K fertilization in 2016-17 and 2017-18.

Treatments	Chlorophyll *a* (mg g^−1^ FW)		Chlorophyll *b* (mg g^−1^ FW)		Carotenoids (mg g^−1^ FW)		Total Chlorophyll (mg g^−1^ FW)	
	2016-17	2017-18	2016-17	2017-18	2016-17	2017-18	2016-17	2017-18
N_23.918_ P_23.918_ K_23.918_	1.08	1.09	0.43	0.44	0.37	0.36	1.68	1.81
N_23.918_ P_23.918_ K_6.082_	1.04	1.32	0.45	0.63	0.33	0.50	1.63	2.01
N_23.918_ P_6.082_ K_23.918_	1.09	1.30	0.45	0.62	0.37	0.49	1.65	1.79
N_23.918_ P_6.082_ K_6.082_	1.13	1.31	0.48	0.58	0.40	0.47	1.72	1.91
N_6.082_ P_23.918_ K_23.918_	0.72	0.82	0.27	0.29	0.26	0.27	1.11	1.22
N_6.082_ P_23.918_ K_6.082_	0.67	0.84	0.24	0.30	0.23	0.31	1.05	1.14
N_6.082_ P_6.082_ K_23.918_	0.74	0.83	0.27	0.32	0.27	0.28	1.01	1.10
N_6.082_ P_6.082_ K_6.082_	0.67	0.97	0.24	0.36	0.25	0.31	0.93	1.01
N_30_ P_15_ K_15_	1.32	1.20	0.62	0.51	0.49	0.42	2.00	1.89
N_0_ P_15_ K_15_	0.67	0.71	0.27	0.26	0.29	0.27	0.97	1.00
N_15_ P_30_ K_15_	1.08	1.16	0.43	0.51	0.36	0.45	1.65	1.69
N_15_ P_0_ K_15_	1.08	0.86	0.41	0.32	0.40	0.27	1.47	1.56
N_15_ P_15_ K_30_	0.88	0.97	0.33	0.38	0.29	0.34	1.44	1.49
N_15_ P_15_ K_0_	1.02	1.12	0.39	0.44	0.38	0.39	1.43	1.47
N_15_ P_15_ K_15_	0.94	1.29	0.35	0.58	0.31	0.46	1.50	1.63
N_15_ P_15_ K_15_	1.09	1.02	0.43	0.40	0.36	0.34	1.55	1.52
N_15_ P_15_ K_15_	1.14	1.10	0.46	0.43	0.37	0.36	1.63	1.62
N_15_ P_15_ K_15_	1.03	1.07	0.42	0.41	0.40	0.35	1.56	1.55
N_15_ P_15_ K_15_	1.02	1.15	0.40	0.47	0.34	0.44	1.51	1.61
N_15_ P_15_ K_15_	1.17	1.16	0.47	0.49	0.41	0.45	1.64	1.68
N_15_ P_15_ K_15_	1.22	1.28	0.54	0.56	0.44	0.46	1.68	1.70
N_15_ P_15_ K_15_	1.10	1.00	0.43	0.37	0.36	0.35	1.64	1.54
N_15_ P_15_ K_15_	1.34	1.12	0.70	0.45	0.50	0.36	1.67	1.66
N_0_ P_0_ K_0_	0.66	0.70	0.21	0.25	0.23	0.25	0.86	0.93

**Table 5:** Regression equation parameter coefficients (Y = a_0_ + a_1_x_1_ + a_2_x_2_ + a_3_x_3_+ a_4_x_1_x_2_ + a_5_x_1_x_3_ + a_6_x_2_x_3_ + a_7_x_1_^2^ + a_8_x_2_^2^ + a_9_x_3_^2^) for MDA, EL, and H_2_O_2_ during 2016-17 and 2017-18.

Y	Year	a_0_	a_1_	a_2_	a_3_	a_4_	a_5_	a_6_	a_7_	a_8_	a_9_	R^2^
MDA	2016-17	0.57	0.15	0.08	-0.02	0.00	0.00	-0.09	-0.17	-0.05	-0.12	0.76**
	2017-18	0.63	0.16	0.03	0.02	0.04	-0.01	-0.09	-0.15	-0.08	-0.11	0.84**
EL	2016-17	0.52	0.18	-0.04	0.02	-0.02	0.06	0.03	-0.07	0.01	0.01	0.58NS
	2017-18	0.53	0.15	0.01	-0.03	-0.02	0.06	-0.05	-0.07	0.06	0.03	0.54NS
H_2_O_2_	2016-17	0.64	0.18	0.06	-0.01	-0.01	0.02	-0.07	-0.16	-0.04	-0.11	0.78**
	2017-18	0.70	0.18	0.03	0.01	0.03	0.01	-0.09	-0.15	-0.06	-0.10	0.87**

**Significance at P < 0.01. * Significance at P < 0.05. NS, Not Significant.

**Table 6:** Regression equation parameter coefficients (Y = a_0_ + a_1_x_1_ + a_2_x_2_ + a_3_x_3_+ a_4_x_1_x_2_ + a_5_x_1_x_3_ + a_6_x_2_x_3_ + a_7_x_1_^2^ + a_8_x_2_^2^ + a_9_x_3_^2^) for soluble protein, SOD, POD, CAT, and proline during 2016-17 and 2017-18.

Y	Year	a_0_	a_1_	a_2_	a_3_	a_4_	a_5_	a_6_	a_7_	a_8_	a_9_	R^2^
Soluble protein	2016-17 2017-18	1.35	0.32	0.09	-0.11	0.11	0.05	0.05	0.07	0.04	0.07	0.57NS
		1.41	0.32	0.13	-0.08	0.10	0.02	-0.03	-0.05	0.03	-0.03	0.65*
SOD	2016-17 2017-18	133.17	41.09	8.99	-9.27	12.49	2.25	7.78	7.00	4.74	7.26	0.67*
		150.08	38.94	13.00	-7.63	5.22	8.63	-2.17	-7.32	1.50	-4.28	0.66*
POD	2016-17 2017-18	67.40	16.93	5.35	-4.95	4.81	1.10	1.67	2.93	1.67	5.20	0.54NS
		70.66	16.98	5.99	-3.69	3.64	0.76	-0.27	-2.54	1.03	-1.80	0.57NS
CAT	2016-17 2017-18	14.64	5.40	-2.18	1.03	-2.01	0.41	0.21	-3.84	-2.58	-1.42	0.88**
		12.40	5.25	-1.87	0.50	-2.33	0.71	-1.15	-2.16	-1.57	0.91	0.79**
Proline	2016-17	60.75	6.54	1.70	-1.26	0.86	-1.13	-0.59	-0.98	-4.39	3.78	0.54NS
	2017-18	53.31	8.09	0.97	-1.05	2.67	-1.45	-1.95	1.57	3.20	6.63	0.72*

**Significance at P < 0.01. * Significance at P < 0.05. NS, Not Significant.

**Table 7:** Regression equation parameter coefficients (Y = a_0_ + a_1_x_1_ + a_2_x_2_ + a_3_x_3_+ a_4_x_1_x_2_ + a_5_x_1_x_3_ + a_6_x_2_x_3_ + a_7_x_1_^2^ + a_8_x_2_^2^ + a_9_x_3_^2^) for chlorophyll *a*, *b*, total chlorophyll, and carotenoids during 2016-17 and 2017-18.

Y	Year	a_0_	a_1_	a_2_	a_3_	a_4_	a_5_	a_6_	a_7_	a_8_	a_9_	R^2^
Chlorophyll *a*	2016-17 2017-18	1.01	0.19	-0.01	-0.01	-0.01	-0.02	0.01	-0.07	-0.04	-0.08	0.81**
		1.07	0.18	0.01	-0.05	-0.01	-0.01	-0.01	-0.05	-0.03	-0.02	0.74*
Chlorophyll *b*	2016-17 2017-18	0.41	0.10	0.00	-0.01	-0.01	-0.01	0.00	-0.02	-0.03	-0.05	0.68*
		0.44	0.10	0.01	-0.02	-0.01	-0.01	-0.03	-0.02	-0.01	-0.01	0.66*
Carotenoids	2016-17 2017-18	0.36	0.06	-0.01	-0.01	-0.01	-0.01	0.01	-0.01	-0.02	-0.03	0.63NS
		0.38	0.07	0.01	-0.02	-0.01	-0.01	-0.02	-0.01	-0.01	-0.01	0.63NS
Total chlorophyll	2016-17 2017-18	1.48	0.32	0.03	0.01	-0.04	-0.02	0.01	-0.07	-0.04	-0.09	0.94**
		1.55	0.33	0.04	-0.01	-0.02	-0.06	-0.01	-0.06	0.00	-0.05	0.95**

**Significance at P < 0.01. * Significance at P < 0.05. NS, Not Significant.

### 3.1 Influence of N, P, and K application on MDA, EL, and H_2_O_2_ contents under cold stress

The MDA, EL, and H_2_O_2_ are important indicators to assess cell membrane integrity and oxidative damage caused by lipid peroxidation. The results indicated that cold stress in the absence of fertilization considerably increased MDA, EL, and H_2_O_2_ (**Table 2**), however, N, P, and K fertilization reduced MDA, EL, and H_2_O_2_ in overseeded perennial ryegrass leaves. For regression equations and R^2^, the data in **Table 1** was transformed (**Supplementary Tables S2**, **S3**) according to the design of the experiment.

Under the cold stress, N, P, and K application reduced the damage caused by oxidative stress through curtailing the levels of MDA by 68% and 57% as compared to control during the two years (**Table 2**). The positive values of regression coefficients a_1_ (N) and a_2_ (P) during the first year and a_1_ (N), a_2_ (P), and a_3_ (K) during the second year indicated amelioration of lipid peroxidation (**Table 5**).

As compared to the control, fertilization under cold stress decreased EL levels by 13% and 12% respectively during the two studies (**Table 2**). The regression coefficients, a_1_ (N) during both years, a_2_ (P), and a_3_ (K) during the second and first year respectively showed positive values (**Table 5**), indicating the mitigation of cell membrane damage by all three nutrients.

Similarly, the production of H_2_O_2_ in response to fertilization under cold stress was curtailed by 32% and 27% during two years as compared to control (**Table 2**). Regarding regression coefficients, a_1_ (N) and a_2_ (P) were positive during both years, while a_3_ (K) was positive during the second year (**Table 5**), suggesting that cold stress-induced H_2_O_2_ was alleviated by these nutrients.

### 3.2 Soluble protein, enzymatic antioxidants (SOD, POD, and CAT), and proline content

Soluble protein, SOD, POD, CAT, and proline in perennial ryegrass were leaves significantly increased by N, P, and K fertilization under cold stress (**Table 3**). Generally, all fertilization treatments alleviated cold-induced adversities by increasing enzymatic and non-enzymatic antioxidant activities as compared to control, with N being the most significant.

N, P, and K treatments combination regulated the activities of soluble protein by 327% and 449%; SOD by 364% and 451%; POD by 340% and 461%; CAT by 488% and 483%; and proline by 77% and 128% under cold stress, respectively, compared with control (**Table 3**).

The regression coefficients for soluble protein, SOD, POD, and proline were positive for a_1_ (N) and a_2_ (P), while negative for a_3_ (K) during both years (**Table 6**). These effects were greatly enhanced by N fertilization as compared to P, leading to more than 3.5-fold and 2.5-fold for soluble protein; 4.5-fold and 3.0-fold for SOD; 3.1-fold and 2.8-fold for POD; 3.8-fold and 8.3-fold for proline, respectively, during two years (**Table 3**). In contrast, CAT activities exhibited positive coefficient values for a_1_ (N) and a_3_ (K) during both years, while negative for a_2_ (P) (**Table 6**).

### 3.3 Photosynthetic pigments (Chl *a*, Chl *b*, T.Chl, and Car)

In our study, in the absence of N, P, and K supply (especially N and P), the cold stress significantly decreased photosynthetic pigments in perennial ryegrass leaves, however, different treatment combinations of N, P, and K enhanced photosynthetic pigments (**Table 4**).

Under cold stress, N, P, and K treatment combinations enhanced Chl *a* content by 103% and 89%, Chl *b* content by 233% and 152%, T.Chl content by 117% and 100%, and Car by 233% and 116%, respectively, during two years as compared with control (**Table 4**).

Regression coefficients of Chl *a* and Car showed positive values for a_1_ (N) during both years and a_2_ (P) only during the first year, while Chl *b* showed positive values for a_1_ (N) and a_2_ (P) whereas negative for a_3_ (K) during both years. The effect of N was greater than P, indicating the larger effect of N, while negative values of K showed non-significant effects (**Table 7**). The positive values of regression coefficients a_1_ (N) and a_2_ (P), and a_3_ (K) in 2016-17 and a_1_ (N) and a_2_ (P) in 2017-18 indicated significant effects with N being the most effective (**Table 7**).

### 3.4 Correlation analysis

**Figure 2:**
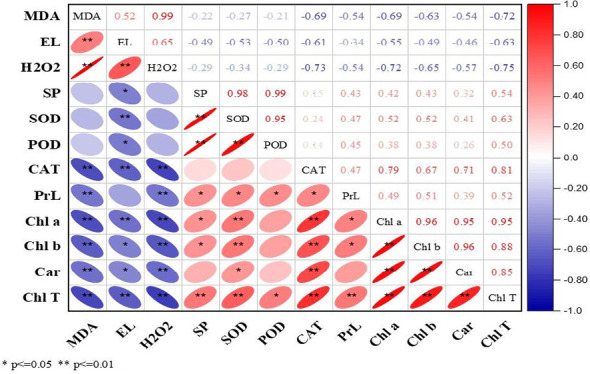
Correlation analysis among studied parameters during 2016-17. The Red and blue color represent the positive and negative correlation. The size and intensity of color exhibited the significance of variables. MDA, malondialdehyde; EL, electrolyte leakage; H_2_O_2_, hydrogen peroxide; SP, soluble protein; SOD, superoxide dismutase; POD, peroxide dismutase; CAT, catalase; PrL, proline; Chl a, chlorophyll a; Chl b, chlorophyll b; Car, carotenoids; Chl T, total chlorophyll.

**Figure 3:**
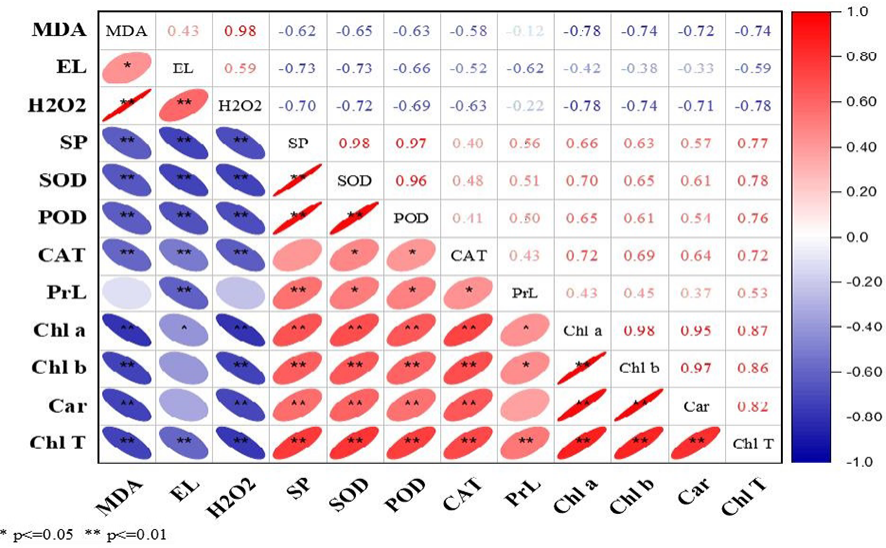
Correlation analysis among studied parameters during 2017-18. The Red and blue color represent the positive and negative correlation. The size and intensity of color exhibited the significance of variables. MDA, malondialdehyde; EL, electrolyte leakage; H_2_O_2_, hydrogen peroxide; SP, soluble protein; SOD, superoxide dismutase; POD, peroxide dismutase; CAT, catalase; PrL, proline; Chl a, chlorophyll a; Chl b, chlorophyll b; Car, carotenoids; Chl T, total chlorophyll.

The current study correlation analysis demonstrated significant results during both years. The ROS (MDA, EL, and H_2_O_2_) were positively correlated with each other, while in contrast, enzymatic and non-enzymatic antioxidants (SOD, POD, CAT, proline), soluble protein, and photosynthetic pigments (Chl *a*, Chl *b*, Car, and T.Chl) were positively correlated with each other (**Figures 2**, **3**). At the same time, ROS (MDA, EL, and H_2_O_2_) exhibited negative correlations with soluble protein, SOD, POD, CAT, Chl *a*, Chl *b*, Car, and T.Chl. This shows that soluble protein, SOD, POD, CAT, and photosynthetic pigments (Chl *a*, Chl *b*, Car, and T.Chl) played a major role in scavenging the ROS (MDA, EL, H_2_O_2_) in overseeded perennial ryegrass thereby counteracting the oxidative damage.

## 4 Discussion

Under natural circumstances in the field, plants often experience several types of environmental stresses on a regular basis. These stresses cause morpho-physiological and biochemical alterations in plants and adversely affect growth and performance (Hasanuzzaman et al., 2020). Extreme low and high temperatures are anticipated to become more frequent (Uleberg et al., 2014; IPCC, 2021) and will cause serious problems for crop production including turfgrasses to perform better specifically in TCZ.

Perennial ryegrass being a cool-season turfgrass and overseeding species is highly sensitive to extremely low temperature, particularly at establishment stages (Guan, 2014; Fan et al., 2020; Xie et al., 2020). Besides cold stress, an optimum supply of macronutrients particularly N, P, and K is imperative for normal plants functioning and coping with stress, while their surfeit or deficiency is detrimental and challenges the survival of plants (Tantray et al., 2020; Kumar et al., 2021; Seleiman et al., 2021). Perennial ryegrass also heavily depends on the optimum supply of N, P, and K for maximum growth, performance, and stress tolerance (Głąb et al., 2020; Zanelli et al., 2021). The findings demonstrated that overseeded perennial ryegrass was adversely affected by cold in terms of reduced soluble protein, SOD, POD, CAT, proline, Chl *a*, Chl *b*, Car, and T.Chl contents and higher MDA, EL, and H_2_O_2_ concentrations. However, N, P, and K application contributed positively to stress tolerance which indicates the importance of these macronutrients.

### 4.1 MDA, EL, and H_2_O_2_

Abiotic stresses cause excessive ROS production that causes the oxidation of proteins, lipids, and nucleic acids which ultimately creates oxidative stress (Hasanuzzaman et al., 2019; Imran et al., 2020; Ihtisham et al., 2021). Similarly, in the present study, cold stress triggered the formation of MDA, EL, and H_2_O_2_ under control conditions and deprivation of any mineral nutrient particularly N (**Table 2**). MDA is a product of the peroxidation of lipid membranes in cells and is used as an indicator of oxidative stress in plants. The increased levels of H_2_O_2_ coincided with higher MDA rates (**Table 2**), which is characterized as a biochemical indicator for free radical-mediated damage in plants under stress. N, P, and K play key roles in various biochemical, enzymatic, non-enzymatic, and metabolic activities, as well as serve as the structural components of many plant compounds and increase plant growth and vigor (Singh et al., 2015; Ma et al., 2022). Our results also illustrated that the application of N, P, and K decreased oxidative stress indicators while on the other hand increasing the activities of enzymatic and non-enzymatic antioxidants such as SOD, POD, CAT, soluble protein, proline, and chloroplastic pigments. Our findings are supported by many earlier studies, that observed increased MDA, EL, and H_2_O_2_ production under cold stress (Jan et al., 2018; Fan et al., 2020; Liu et al., 2020; Su et al., 2021; Ma et al., 2022), N deprivation (Tantray et al., 2020), P deprivation (Noor et al., 2021), and K deprivation (Seleiman et al., 2021). Higher accumulation of MDA, EL, and H_2_O_2_ under N deprivation as compared with P and K was corroborated by earlier studies (Tewari et al., 2007; Hussain et al., 2016).

### 4.2 Soluble protein, SOD, POD, CAT, and proline content

Plants have evolved a complex system of enzymatic and non-enzymatic antioxidants which increases during stress conditions to cope with stress-induced adversities. In this study, cold stress aggravated oxidative damage in overseeded perennial ryegrass, while N, P, and K fertilization especially N and P attenuated that damage, which can be attributed to the accumulation of enhanced antioxidants enzymatic activities (**Tables 3**, **6**). Soluble protein and both enzymatic (e.g., SOD, POD, and CAT) and non-enzymatic (e.g., proline) antioxidants played key roles in scavenging the ROS in perennial ryegrass thereby counteracting the oxidative damage. These antioxidant activities are considered to curb the cascades of unrestricted oxidation and defend ROS-induced oxidative damage to the plant cells. The enhanced rates of antioxidants confer capability to the plants to scavenge ROS and resist cold stress (Anjum et al., 2015; Moustafa-Farag et al., 2020; Hasanuzzaman et al., 2021; Imran et al., 2021). Generally, ROS is scavenged by antioxidant enzymes, e.g., proteins deoxidize lipids and nucleic acids (Mittler, 2002), SOD contributes to catalysis of the dismutation of superoxide (O_2_^•−^) (Fahad et al., 2016), whereas the POD and CAT play important roles in scavenging of H_2_O_2_ (Sachdev et al., 2021). The role of proline is variable, it acts as a radical scavenger in plants under stress as well as an osmotic agent (Hayat et al., 2012). Increased levels of proline attenuate the damage caused by stress in plant cells by adjusting osmotic potential and reducing water potential (Imran et al., 2020). The elevated antioxidant activities in N, P, and K treated plots particularly N and P correspond to better ROS scavenging ability and protecting cell membrane integrity that conferred tolerance against cold stress. N is an essential part of proteins and amino acids that helps in enzyme regulation, P is the part of important molecules at cellular levels (like, ATP, ADP, NADPH, and nucleicacids), while K is inevitable for plant growth and development, and contribute to enzyme catalysis, charge balance, and osmotic adjustment that eventually attenuated cold stress adversities (Hussain et al., 2016; Kumar et al., 2021). N in the form of nitric oxide is a highly reactive, membrane-permanent free radical with a broad spectrum of regulatory functions in several physiological processes and protects the plant against stress by acting as an antioxidant directly scavenging the ROS generated under cold stress (Wendehenne et al., 2001). K is essential for many physiological processes, such as photosynthesis, translocation of photosynthates into sink organs, maintenance of turgidity, and activation of enzymes under stress conditions (Mengel and Kirkby, 2001). Plants have developed a wide range of adaptive/resistance mechanisms to maintain productivity and ensure survival under a variety of environmental stresses, including cold stress, which affects the fluidity of membrane lipids and thus may alter membrane structure (Marschner, 1995). Under lower amounts of K, cold-induced photo-oxidative damage can be exacerbated causing a decrease in plant growth and yield while adequate K application can provide protection against oxidative damage caused by cold stress (Waraich et al., 2012). The enhanced levels of these enzymatic activities by N, P, and K can also be correlated with decreased levels of MDA, EL, and H_2_O_2_ as shown by correlation analysis (**Figures 2**, **3**). Similar results were reported by (Abid et al., 2016; Shao et al., 2020) that N fertilization conferred tolerance to stress conditions by maintaining higher enzymatic and non-enzymatic anti-oxidants and lower ROS generation. Results with increased enzymatic activities under stress conditions in response to N, P, and K are recently reported by Ma et al. (2022). Corresponding results with N fertilization (Chang et al., 2016; Ahanger et al., 2019), P fertilization (Noor et al., 2021), and K fertilization (Seleiman et al., 2021) have been previously reported.

### 4.3 Photosynthetic pigments

Our results revealed that cold stress significantly reduced the photosynthetic pigments in overseeded perennial ryegrass while fertilization on the other hand highly increased photosynthetic pigments (**Tables 4**, **7**). The effects were so obvious that necrosis in the leaves occurred under control conditions and zero N. The enhanced chloroplastic pigments in response to N, P, and K, particularly N and then P were correlated with increased turf color, total protein, and photosynthesis in previous studies (Ihtisham et al., 2018; Jena and Mohanty, 2020; Ihtisham et al., 2021). N fertilization promotes the accumulation of chlorophyll as it is the major component of chlorophyll (Raza et al., 2022; Roy et al., 2022). Certainly, deficient-N plants show deficient-chlorophyll phenotypes. P and K are not chlorophyll components, the increase recorded in chloroplastic pigments under P might be the result of higher photosynthesis, whereas for K the increase is probably due to secondary mechanisms associated with general growth promotion. The correlation between chloroplastic pigments and photosynthesis can also be justified, as chlorophyll serves as the key light-harvesting pigment for photosystem, and accumulate extremely high levels of photosynthetic proteins, like light-harvesting chlorophyll a/b-binding protein and Rubisco (Ihtisham et al., 2018). N, being a key component of proteins, chlorophyll, and Rubisco affected the whole metabolism of plants during cold stress. Reduced chlorophyll contents in turfgrasses under limited N fertilization and abiotic stress conditions were also previously reported by (Shen et al., 2020; Januškaitienė et al., 2021). The enhanced levels of photosynthetic pigments in response to N, P, and K could also be ascribed to the regulation of enzymatic and non-enzymatic antioxidants defense systems and decreased lipid peroxidation, cell membrane damage, and H_2_O_2_ in this study, as confirmed by the correlation analysis. Similarly, previous studies reported that N, P, and K application under stress conditions actively synthesized chlorophyll contents, which was verified by higher chloroplastic pigments, increased antioxidants activities, and lower ROS production (Kozłowska et al., 2021; Saleem et al., 2021; Xu et al., 2021).

## 5 Conclusion

Exogenous applications of N, P, and K are considered an effective way to attenuate plant’s cold stress. The results of the present study elucidated that the absence of N, P, and K fertilization as evident from the controlled conditions under cold stress and TCZ triggered lipid peroxidation, cell membrane damage, and excessive hydrogen peroxide accumulation presumably by desynchronizing the ROS-scavenging mechanism in overseeded perennial ryegrass. Nevertheless, N, P, and K application proficiently relieved cold stress provoked inhibitory effects in overseeded perennial ryegrass which could mainly be ascribed to decreased MDA, EL, and H_2_O_2_ contents, while increased enzymatic and non-enzymatic antioxidants (soluble protein, SOD, POD, CAT, and proline) and photosynthetic pigments (Chl *a*, Chl *b*, T.Chl, and Car) through elevated ROS scavenging. Thus, our results conclude that NPK application maximized the performance and cold stress tolerance of perennial ryegrass that was overseeded on dormant bermudagrass under a transitional climate. To cope with cold stress, turfgrass management will depend not only on breeding new varieties that are well adapted to cold climates but also on the development of nutrient management strategies, that will contribute to sustainable agriculture.

## Data availability statement

The original contributions presented in the study are included in the article/**Supplementary Material**. Further inquiries can be directed to the corresponding author.

## Author contributions

MI conceived the idea of the experiment and wrote the manuscript. MH provided supervision and technical guidance and revised the manuscript. FZ, NK, and AR contributed to data processing, figures, and tables. MS, SK, AE-S, MA, and MJH helped in statistical analysis, technical guidance, general support, and formatting. JL, XMZ, and XZ provided supervision and funding. All authors contributed to the article and approved the submitted version.

## References

[B1] Abid M. Tian Z. Ata-Ul-Karim S. T. Cui Y. Liu Y. Zahoor R.. (2016). Nitrogen nutrition improves the potential of wheat (*Triticum aestivum* l.) to alleviate the effects of drought stress during vegetative growth periods. Front. Plant Sci. 7. doi: 10.3389/fpls.2016.00981 PMC4927619 27446197

[B2] Ahanger M. A. Qin C. Begum N. Maodong Q. Dong X. X. El-Esawi M.. (2019). Nitrogen availability prevents oxidative effects of salinity on wheat growth and photosynthesis by up-regulating the antioxidants and osmolytes metabolism, and secondary metabolite accumulation. BMC Plant Biol. 19, 1–12. doi: 10.1186/s12870-019-2085-3 31703619 PMC6839093

[B3] Anjum S. A. Tanveer M. Hussain S. Bao M. Wang L. Khan I.. (2015). Cadmium toxicity in maize (*Zea mays* l.): Consequences on antioxidative systems, reactive oxygen species and cadmium accumulation. Environ. Sci. pollut. Res. 22, 17022–17030. doi: 10.1007/s11356-015-4882-z 26122572

[B4] Atayee A. R. Noori M. S. (2020). Alleviation of cold stress in vegetable crops. J. Sci. Agric. 4, 38–44. doi: 10.25081/jsa.2020.v4.6110

[B5] Barker L. J. Hannaford J. Parry S. Smith K. A. Tanguy M. Prudhomme C. (2019). Historic hydrological droughts 1891–2015: Systematic characterisation for a diverse set of catchments across the UK. Hydrol. Earth Syst. Sci. Discuss 23, 4583–4602. doi: 10.5194/hess-23-4583-2019

[B6] Bates L. S. Waldren R. P. Teare I. (1973). Rapid determination of free proline for water-stress studies. Plant Soil 39, 205–207. doi: 10.1007/BF00018060

[B7] Bera K. Dutta P. Sadhukhan S. (2022). “Plant responses under abiotic stress and mitigation options towards agricultural sustainability,” In: Roy S Mathur P Chakraborty AP Saha SP editors. Plant stress: challenges and management in the new decade. (Cham, Switzerland: Advances in Science, Technology & Innovation). p. 3–28. doi: 10.1007/978-3-030-95365-2_1

[B8] Chang Z. Liu Y. Dong H. Teng K. Han L. Zhang X. (2016). Effects of cytokinin and nitrogen on drought tolerance of creeping bentgrass. PloS One 11, e0154005. doi: 10.1371/journal.pone.0154005 27099963 PMC4839601

[B9] Christians N. E. Patton A. J. Law Q. D. (2016). Fundamentals of turfgrass management (Hoboken, NJ: John Wiley & Sons, Inc). doi: 10.1002/9781119308867

[B10] Dahro B. Wang F. Peng T. Liu J. (2016). PtrA/NINV, an alkaline/neutral invertase gene of *Poncirus trifoliata*, confers enhanced tolerance to multiple abiotic stresses by modulating ROS levels and maintaining photosynthetic efficiency. BMC Plant Biol. 16, 1–18. doi: 10.1186/s12870-016-0761-0 27025596 PMC4812658

[B11] El-Sappah A. H. Abbas M. Rather S. A. Wani S. H. Soaud N. Noor Z.. (2023). Genome-wide identification and expression analysis of metal tolerance protein (MTP) gene family in soybean (*Glycine max*) under heavy metal stress. Mol. Biol. Rep. 18: 1–16. doi: 10.1007/s11033-022-08100-x 36653731

[B12] El-Sappah A. H. Elbaiomy R. G. Elrys A. S. Wang Y. Zhu Y. Huang Q.. (2021a). Genome-wide identification and expression analysis of metal tolerance protein gene family in *Medicago truncatula* under a broad range of heavy metal stress. Front. Genet. 12. doi: 10.3389/fgene.2021.713224 PMC8482800 34603378

[B13] El-Sappah A. H. Elrys A. S. Desoky E.-S. M. Zhao X. Bingwen W. El-Sappah H. H.. (2021b). Comprehensive genome wide identification and expression analysis of MTP gene family in tomato (*Solanum lycopersicum*) under multiple heavy metal stress. Saudi J. Biol. Sci. 28, 6946–6956. doi: 10.1016/j.sjbs.2021.07.073 34866994 PMC8626246

[B14] El-Sappah A. H. Rather S. A. (2022). “Genomics approaches to study abiotic stress tolerance in plants,” in Plant abiotic stress physiology: Volume 2: Molecular advancements, (Burlington: Apple Academic Press) vol. 2, 25. doi: 10.1201/9781003180579-2

[B15] Fahad S. Hussain S. Saud S. Hassan S. Tanveer M. Ihsan M. Z.. (2016). A combined application of biochar and phosphorus alleviates heat-induced adversities on physiological, agronomical and quality attributes of rice. Plant Physio. Bioch. 103, 191–198. doi: 10.1016/j.plaphy.2016.03.001 26995314

[B16] Faiq M. H. Noori M. S. (2021). Utilization of phytohormones for successful crop production under environmental stress conditions. J. Sci. Agric. 5, 60–66. doi: 10.25081/jsa.2021.v5.7281

[B17] Fan J. Ren J. Zhu W. Amombo E. Fu J. Chen L. (2014). Antioxidant responses and gene expression in bermudagrass under cold stress. Hortic. Sci. 139, 699–705. doi: 10.21273/JASHS.139.6.699

[B18] Fan J. Zhang W. Amombo E. Hu L. Kjorven J. O. Chen L. (2020). Mechanisms of environmental stress tolerance in turfgrass. Agronomy 10, 522. doi: 10.3390/agronomy10040522

[B19] Głąb T. Szewczyk W. Gondek K. Mierzwa-Hersztek M. Palmowska J. Nęcka K. (2020). Optimization of turfgrass fertigation rate and frequency. Agr. Water Manage. 234, 106107. doi: 10.1016/j.agwat.2020.106107

[B20] Guan X. (2014). Physiology of cold acclimation and deacclimation responses of cool-season grasses: Carbon and hormone metabolism. Masters Theses 2014: 130. doi: 10.7275/6042177

[B21] Hasanuzzaman M. Bhuyan M. Anee T. I. Parvin K. Nahar K. Mahmud J. A.. (2019). Regulation of ascorbate-glutathione pathway in mitigating oxidative damage in plants under abiotic stress. Antioxidants 8, 384. doi: 10.3390/antiox8090384 31505852 PMC6770940

[B22] Hasanuzzaman M. Bhuyan M. Zulfiqar F. Raza A. Mohsin S. M. Mahmud J. A.. (2020). Reactive oxygen species and antioxidant defense in plants under abiotic stress: Revisiting the crucial role of a universal defense regulator. Antioxidants 9, 681. doi: 10.3390/antiox9080681 32751256 PMC7465626

[B23] Hasanuzzaman M. Inafuku M. Nahar K. Fujita M. Oku H. (2021). Nitric oxide regulates plant growth, physiology, antioxidant defense, and ion homeostasis to confer salt tolerance in the mangrove species, *Kandelia obovata* . Antioxidants 10, 611. doi: 10.3390/antiox10040611 33923816 PMC8073094

[B24] Hayat S. Hayat Q. Alyemeni M. N. Wani A. S. Pichtel J. Ahmad A. (2012). Role of proline under changing environments: A review. Plant Signal. Behav. 7, 1456–1466. doi: 10.4161/psb.21949 22951402 PMC3548871

[B25] Hoffman L. DaCosta M. Ebdon J. S. Watkins E. (2010). Physiological changes during cold acclimation of perennial ryegrass accessions differing in freeze tolerance. Crop Sci. 50, 1037–1047. doi: 10.2135/cropsci2009.06.0293

[B26] Hu L. Bi A. Hu Z. Amombo E. Li H. Fu J. (2018). Antioxidant metabolism, photosystem II, and fatty acid composition of two tall fescue genotypes with different heat tolerance under high temperature stress. Front. Plant Sci. 9. doi: 10.3389/fpls.2018.01242 PMC6113381 30186304

[B28] Huang S. Jiang S. Liang J. Chen M. Shi Y. (2019). Current knowledge of bermudagrass responses to abiotic stresses. Breed. Sci. 69, 215–226. doi: 10.1270/jsbbs.18164 31481830 PMC6711739

[B27] Huang C. Qin N. Sun L. Yu M. Hu W. Qi Z. (2018). Selenium improves physiological parameters and alleviates oxidative stress in strawberry seedlings under low-temperature stress. Int. J. Mol. Sci. 19 (7), 1913. doi: 10.3390/ijms19071913 29966265 PMC6073314

[B29] Huang X. Shi H. Hu Z. Liu A. Amombo E. Chen L.. (2017). ABA is involved in regulation of cold stress response in bermudagrass. Front. Plant Sci. 8. doi: 10.3389/fpls.2017.01613 PMC5645512 29081782

[B30] Hussain S. Khan F. Cao W. Wu L. Geng M. (2016). Seed priming alters the production and detoxification of reactive oxygen intermediates in rice seedlings grown under sub-optimal temperature and nutrient supply. Front. Plant Sci. 7. doi: 10.3389/fpls.2016.00439 PMC4820636 27092157

[B31] Ihtisham M. Fahad S. Luo T. Larkin R. M. Yin S. Chen L. (2018). Optimization of nitrogen, phosphorus, and potassium fertilization rates for overseeded perennial ryegrass turf on dormant bermudagrass in a transitional climate. Front. Plant Sci. 9. doi: 10.3389/fpls.2018.00487 PMC5911507 29713331

[B32] Ihtisham M. Liu S. Shahid M. O. Khan N. Lv B. Sarraf M.. (2020). The optimized n, p, and K fertilization for bermudagrass integrated turf performance during the establishment and its importance for the sustainable management of urban green spaces. Sustainability 12, 10294. doi: 10.3390/su122410294

[B33] Ihtisham M. Noori A. Yadav S. Sarraf M. Kumari P. Brestic M.. (2021). Silver nanoparticle’s toxicological effects and phytoremediation. Nanomaterials 11, 2164. doi: 10.3390/nano11092164 34578480 PMC8465113

[B34] Imran M. Hussain S. El-Esawi M. A. Rana M. S. Saleem M. H. Riaz M.. (2020). Molybdenum supply alleviates the cadmium toxicity in fragrant rice by modulating oxidative stress and antioxidant gene expression. Biomolecules 10, 1582. doi: 10.3390/biom10111582 33233373 PMC7700372

[B35] Imran M. Hussain S. He L. Ashraf M. F. Ihtisham M. Warraich E. A.. (2021). Molybdenum-induced regulation of antioxidant defense-mitigated cadmium stress in aromatic rice and improved crop growth, yield, and quality traits. Antioxidants 10, 838. doi: 10.3390/antiox10060838 34073960 PMC8225192

[B36] IPCC (2021). “Climate change: The Physical Science Basis,” Contribution of Working Group I to the Sixth Assessment Report of the Intergovernmental Panel on Climate Change. Masson-Delmotte V. Zhai P. Pirani A. Connors S.L. Péan C. Berger S. (Eds) (Cambridge, United Kingdom and New York, NY, USA: Cambridge University Press) 2391 pp. doi: 10.1017/9781009157896

[B37] Jan N. Majeed U. Andrabi K. I. John R. (2018). Cold stress modulates osmolytes and antioxidant system in *Calendula officinalis* . Acta Physiol. Plant 40, 1–16. doi: 10.1007/s11738-018-2649-0

[B38] Januškaitienė I. Kacienė G. Dikšaitytė A. Žaltauskaitė J. Miškelytė D. Sujetovienė G.. (2021). Nitrogen supplement attenuates drought stress for non-leguminous hybrid plant fescue and does not affect nitrogen-fixing alfalfa. J. Agron 208:3, 283-294. doi: 10.1111/jac.12576

[B39] Jena K. Mohanty C. (2020). Effect of nitrogen and phosphorus on growth and quality of Bermuda lawn grass (*Cynodon dactylon*) cv. selection-1. Pharm. Innov. J. 9:3, 56–60.

[B40] Johnson R. Vishwakarma K. Hossen M. S. Kumar V. Shackira A. M. Puthur J. T.. (2022). Potassium in plants: Growth regulation, signaling, and environmental stress tolerance. Plant Physiol. Biochem. 172, 56–69. doi: 10.1016/j.plaphy.2022.01.001 35032888

[B41] Kozłowska M. Bandurska H. Breś W. (2021). Response of lawn grasses to salinity stress and protective potassium effect. Agronomy 11, 843. doi: 10.3390/agronomy11050843

[B43] Kumar S. Kumar S. Mohapatra T. (2021). Interaction between macro-and micro-nutrients in plants. Front. Plant Sci. 10; 12:665583. doi: 10.3389/fpls.2021.665583 PMC8141648 34040623

[B42] Kumar D. Punetha A. Verma P. P. Padalia R. C. (2021). Micronutrient based approach to increase yield and quality of essential oil in aromatic crops. Front. Plant Sci. 1; 26: 100361. doi: 10.1016/j.jarmap.2021.100361

[B44] Li Z. Zhang R. Xia S. Wang L. Liu C. Zhang R.. (2019). Interactions between n, p and K fertilizers affect the environment and the yield and quality of satsumas. Glob. Ecol. Conserv. 19, e00663. doi: 10.1016/j.gecco.2019.e00663

[B45] Liu T. Ye X. Li M. Li J. Qi H. Hu X. (2020). H_2_O_2_ and NO are involved in trehalose-regulated oxidative stress tolerance in cold-stressed tomato plants. Environ. Exp. Bot. 171, 103961. doi: 10.1016/j.envexpbot.2019.103961

[B46] Ma J. Ali S. Saleem M. H. Mumtaz S. Yasin G. Ali B.. (2022). Short-term responses of spinach (*Spinacia oleracea* L.) to the individual and combinatorial effects of nitrogen, phosphorus and potassium and silicon in the soil contaminated by boron. Front. Plant Sci. 13, 983156. doi: 10.3389/fpls.2022.983156 36212291 PMC9540599

[B47] Marcum K. B. (1998). Cell membrane thermostability and whole-plant heat tolerance of Kentucky bluegrass. Crop Sci. 38, 1214–1218. doi: 10.2135/cropsci1998.0011183X003800050017x

[B48] Marschner H. (1995). Mineral nutrition of higher plants, 2nd Edn. (London: Academic Press).

[B49] Mengel K. Kirkby E. A. (2001). Principles of plant nutrition. 5th edition (Dordrecht: Kluwer Academic Publishers).

[B50] Mittler R. (2002). Oxidative stress, antioxidants and stress tolerance. Trends Plant Sci. 7, 405–410. doi: 10.1016/S1360-1385(02)02312-9 12234732

[B51] Montgomery D. C. (2001). Design and Analysis of Experiments. (New York, NY: JohnWiley & Sons).

[B52] Moustafa-Farag M. Mahmoud A. Arnao M. B. Sheteiwy M. S. Dafea M. Soltan M.. (2020). Melatonin-induced water stress tolerance in plants: Recent advances. Antioxidants 9, 809. doi: 10.3390/antiox9090809 32882822 PMC7554692

[B53] Noor I. Sohail H. Hasanuzzaman M. Hussain S. Li G. Liu J. (2021). Phosphorus confers tolerance against manganese toxicity in *Prunus persica* by reducing oxidative stress and improving chloroplast ultrastructure. Chemosphere, 132999. doi: 10.1016/j.chemosphere.2021.132999 34808198

[B54] Ostrem L. Rapacz M. Larsen A. Marum P. Rognli O. A. (2018). Chlorophyll a fluorescence and freezing tests as selection methods for growth cessation and increased winter survival in xFestulolium. Front. Plant Sci. 9. doi: 10.3389/fpls.2018.01200 PMC6109792 30177939

[B55] Ozkan S. S. Kir B. (2021). Effects of overseeding times on different warm-season turfgrasses: Visual turf quality and some related characteristics. Ital. J. Agron. 16: 3. doi: 10.4081/ija.2021.1820

[B56] Raza A. Yin C. Asghar M. A. Ihtisham M. Shafiq I. Cheng B.. (2022). Foliar application of NH_4_ ^+^/NO_3_ ^-^ ratios enhance the lodging resistance of soybean stem by regulating the physiological and biochemical mechanisms under shade conditions. Front. Plant Sci. 13. doi: 10.3389/fpls.2022.906537 PMC9353630 35937330

[B57] Roy R. Mostofa M. G. Wang J. Fornara D. Sarker T. Zhang R. (2021). Revegetation intervention of drought-prone coal-mined spoils using *Caragana korshinskii* under variable water and nitrogen-phosphorus resources. Agr. Water Manage. 246, 106712. doi: 10.1016/j.agwat.2020.106712

[B58] Roy R. Sultana S. Wang J. Mostofa M. G. Sarker T. Shah M. M. R.. (2022). Revegetation of coal mine degraded arid areas: The role of a native woody species under optimum water and nutrient resources. Environ. Res. 204, 111921. doi: 10.1016/j.envres.2021.111921 34454933

[B59] Sachdev S. Ansari S. A. Ansari M. I. Fujita M. Hasanuzzaman M. (2021). Abiotic stress and reactive oxygen species: Generation, signaling, and defense mechanisms. Antioxidants 10, 277. doi: 10.3390/antiox10020277 33670123 PMC7916865

[B60] Saleem M. H. Wang X. Ali S. Zafar S. Nawaz M. Adnan M.. (2021). Interactive effects of gibberellic acid and NPK on morpho-physio-biochemical traits and organic acid exudation pattern in coriander (*Coriandrum sativum* l.) grown in soil artificially spiked with boron. Plant Physiol. Biochem. 167, 884–900. doi: 10.1016/j.plaphy.2021.09.015 34537578

[B61] Sarraf M. Kataria S. Taimourya H. Santos L. O. Menegatti R. D. Jain M.. (2020). Magnetic field (MF) applications in plants: An overview. Plants 9, 1139. doi: 10.3390/plants9091139 32899332 PMC7570196

[B62] Seleiman M. F. Al-Suhaibani N. Ali N. Akmal M. Alotaibi M. Refay Y.. (2021). Drought stress impacts on plants and different approaches to alleviate its adverse effects. Plants 10, 259. doi: 10.3390/plants10020259 33525688 PMC7911879

[B63] Shao A. Sun Z. Fan S. Xu X. Wang W. Amombo E.. (2020). Moderately low nitrogen application mitigate the negative effects of salt stress on annual ryegrass seedlings. PeerJ 8, e10427. doi: 10.7717/peerj.10427 33344081 PMC7719293

[B64] Shen H. Dong S. Li S. Wang W. Xiao J. Yang M.. (2020). Effects of warming and N deposition on the physiological performances of *Leymus secalinus* in alpine meadow of qinghai-Tibetan plateau. Front. Plant Sci. 10. doi: 10.3389/fpls.2019.01804 PMC7047333 32153598

[B65] Shi H. Ye T. Zhong B. Liu X. Chan Z. (2014). Comparative proteomic and metabolomic analyses reveal mechanisms of improved cold stress tolerance in bermudagrass (*Cynodon dactylon* (L.) pers.) by exogenous calcium. J. Integr. Plant Biol. 56, 1064–1079. doi: 10.1093/jxb/eru373 24428341

[B66] Singh M. K. Chand T. Kumar M. Singh K. Lodhi S. Singh V.. (2015). Response of different doses of NPK and boron on growth and yield of broccoli (*Brassica oleracea* L. var. italica). Int. J. Bio-Resour. Stress Manage. 6, 108–112. doi: 10.5958/0976-4038.2015.00016.0

[B67] Su Y. Huang Y. Dong X. Wang R. Tang M. Cai J.. (2021). Exogenous methyl jasmonate improves heat tolerance of perennial ryegrass through alteration of osmotic adjustment, antioxidant defense, and expression of jasmonic acid-responsive genes. Front. Plant Sci. 12. doi: 10.3389/fpls.2021.664519 PMC8137847 34025701

[B68] Tantray A. Y. Bashir S. S. Ahmad A. (2020). Low nitrogen stress regulates chlorophyll fluorescence in coordination with photosynthesis and rubisco efficiency of rice. Physiol. Mol. Biol. Plants 26, 83–94. doi: 10.1007/s12298-019-00721-0 32158122 PMC7036394

[B69] Tewari R. K. Kumar P. Sharma P. N. (2007). Oxidative stress and antioxidant responses in young leaves of mulberry plants grown under nitrogen, phosphorus or potassium deficiency. J. Integr. Plant Biol. 49, 313–322. doi: 10.1111/j.1744-7909.2007.00358.x

[B70] Tewari R. K. Yadav N. Gupta R. Kumar P. (2021). Oxidative stress under macronutrient deficiency in plants. J. Soil Sci. Plant Nutr. 21 (1), 832–859. doi: 10.1007/s42729-020-00405-9

[B71] Uleberg E. Hanssen-Bauer I. van Oort B. Dalmannsdottir S. (2014). Impact of climate change on agriculture in northern Norway and potential strategies for adaptation. Climatic Change 122, 27–39. doi: 10.1007/s10584-013-0983-1

[B72] Waraich E. A. Ahmad R. Ashraf M. Y. Saifullah Ahmad M. (2011). Improving agricultural water use efficiency by nutrient management in crop plants. Acta Agriculturae Scandinavica Section B-Soil Plant Sci. 61 (4), 291–304. doi: 10.1080/09064710.2010.491954

[B73] Waraich E. A. Ahmad R. Halim A. Aziz T. (2012). Alleviation of temperature stress by nutrient management in crop plants: A review. J. Soil Sci. Plant Nutr. 12 (2), 221–244. doi: 10.4067/S0718-95162012000200003

[B74] Wendehenne D. Pugin A. Klessig D. F. Durner J. (2001). Nitric oxide: comparative synthesis and signaling in animal and plant cells. Trends Plant Sci. 6 (4), 177–183. doi: 10.1016/S1360-1385(01)01893-3 11286923

[B75] Xie F. Datta R. Qin D. (2020). “Plant growth and morphophysiological modifications in perennial ryegrass under environmental stress,” in Abiotic stress in plants. (London: IntechOpen United Kingdom), 353–369. doi: 10.5772/intechopen.93709

[B76] Xu H.-S. Zhu L. Mei Y. (2021). Effects of high levels of nitrogen and phosphorus on perennial ryegrass (*Lolium perenne* l.) and its potential in bioremediation of highly eutrophic water. Environ. Sci. pollut. Res. 28, 9475–9483. doi: 10.1007/s11356-020-11458-9 33146824

[B77] Yin Y. Deng H. Ma D. (2022). Complex effects of moisture conditions and temperature enhanced vegetation growth in the arid/humid transition zone in northern China. Sci. Total Environ. 805, 150152. doi: 10.1016/j.scitotenv.2021.150152 34543796

[B78] Yin Y. Deng H. Ma D. Wu S. (2021). Intensified risk to ecosystem productivity under climate change in the arid/humid transition zone in northern China. J. Geogr. Sci. 31, 1261–1282. doi: 10.1007/s11442-021-1897-x

[B79] Zaman F. Zhang E. Khattak W. A. Li J. Ilyas M. Deng X.. (2022). Natural variations and dynamics of macronutrients for 87 tea plant (*Camellia sinensis*) varieties throughout the growing seasons in wuhan. Scientia Hortic. 306, 111425. doi: 10.1016/j.scienta.2022.111425

[B80] Zanelli B. Vidrih M. Bohinc T. Trdan S. (2021). Impact of fertilisers on five turfgrass mixtures for football pitches under natural conditions. Hortic. Sci. 48, 190–204. doi: 10.17221/160/2020-HORTSCI

[B81] Zhang Z. Lynch J. P. Zhang B. Wang Q. (2017). “NPK deficiency modulates oxidative stress in plants,” in Plant macronutrient use efficiency (New York, Elsevier Inc.: Academic Press), 245–265. doi: 10.1016/b978-0-12-811308-0.00014-4

