# Supplementary material for: Primary plant nutrients modulate the reactive oxygen species metabolism and mitigate the impact of cold stress in overseeded perennial ryegrass
Source: Front Plant Sci. 2023 Mar 31;14:1149832. doi: 10.3389/fpls.2023.1149832 (PMC10103648; doi:10.3389/fpls.2023.1149832)
Supplement: Supplementary file 1 [file DataSheet_1.docx]

**Supplementary Table S1.** Experimental scheme (design and treatment combinations) of the central composite rotatable design (CCRD).

| **Treatments** | **Coded symbols** | | | **Actual variable levels** | | |
| --- | --- | --- | --- | --- | --- | --- |
|  | **X_1_** | **X_2_** | **X_3_** | **N (g m^-2^)** | **P (g m^-2^)** | **K (g m^-2^)** |
| 1 | 1 | 1 | 1 | 23.918 | 23.918 | 23.918 |
| 2 | 1 | 1 | –1 | 23.918 | 23.918 | 6.082 |
| 3 | 1 | –1 | 1 | 23.918 | 6.082 | 23.918 |
| 4 | 1 | –1 | –1 | 23.918 | 6.082 | 6.082 |
| 5 | –1 | 1 | 1 | 6.082 | 23.918 | 23.918 |
| 6 | –1 | 1 | –1 | 6.082 | 23.918 | 6.082 |
| 7 | –1 | –1 | 1 | 6.082 | 6.082 | 23.918 |
| 8 | –1 | –1 | –1 | 6.082 | 6.082 | 6.082 |
| 9 | 1.682 | 0 | 0 | 30 | 15 | 15 |
| 10 | –1.682 | 0 | 0 | 0 | 15 | 15 |
| 11 | 0 | 1.682 | 0 | 15 | 30 | 15 |
| 12 | 0 | –1.682 | 0 | 15 | 0 | 15 |
| 13 | 0 | 0 | 1.682 | 15 | 15 | 30 |
| 14 | 0 | 0 | –1.682 | 15 | 15 | 0 |
| 15 | 0 | 0 | 0 | 15 | 15 | 15 |
| 16 | 0 | 0 | 0 | 15 | 15 | 15 |
| 17 | 0 | 0 | 0 | 15 | 15 | 15 |
| 18 | 0 | 0 | 0 | 15 | 15 | 15 |
| 19 | 0 | 0 | 0 | 15 | 15 | 15 |
| 20 | 0 | 0 | 0 | 15 | 15 | 15 |
| 21 | 0 | 0 | 0 | 15 | 15 | 15 |
| 22 | 0 | 0 | 0 | 15 | 15 | 15 |
| 23 | 0 | 0 | 0 | 15 | 15 | 15 |
| 24 |  |  |  | 0 | 0 | 0 |

* Treatment 1 to 24 represents total experimental treatments and the actual variable levels of N, P, and K. X_1_, X_2_, X_,3_, indicate coded symbols for N, P, K. T24 represents the control treatment.

**Supplementary Table S2.** Transformed values of data in table 2 according to the design (CCRD) of the experiment using formula: $X=\frac{x - x_{min}}{x_{\max- x_{min}}}$ where $X$ represents transformed value and $x$ represents the observed value of each treatment, and xmax and xmin represent maximum and minimum values observed in table in table 2.

| **Treatments** | **MDA (nmol mg^-1^ protein)** | | **EL (%)** | | **H_2_O_2_ (nmol mg^-1^ protein)** | |
| --- | --- | --- | --- | --- | --- | --- |
|  | 2016-17 | 2017-18 | 2016-17 | 2017-18 | 2016-17 | 2017-18 |
| N_23.918_ P_23.918_ K_23.918_ | 0.46 | 0.36 | 0.24 | 0.24 | 0.34 | 0.24 |
| N_23.918_ P_23.918_ K_6.082_ | 0.34 | 0.16 | 0.37 | 0.26 | 0.25 | 0.06 |
| N_23.918_ P_6.082_ K_23.918_ | 0.43 | 0.22 | 0.00 | 0.25 | 0.25 | 0.12 |
| N_23.918_ P_6.082_ K_6.082_ | 0.49 | 0.42 | 0.29 | 0.43 | 0.37 | 0.34 |
| N_6.082_ P_23.918_ K_23.918_ | 0.84 | 0.74 | 0.65 | 0.69 | 0.77 | 0.69 |
| N_6.082_ P_23.918_ K_6.082_ | 0.53 | 0.62 | 0.79 | 0.41 | 0.53 | 0.52 |
| N_6.082_ P_6.082_ K_23.918_ | 0.64 | 0.52 | 0.75 | 0.71 | 0.62 | 0.50 |
| N_6.082_ P_6.082_ K_6.082_ | 0.87 | 0.68 | 0.52 | 0.69 | 0.76 | 0.63 |
| N_30_ P_15_ K_15_ | 0.41 | 0.40 | 0.52 | 0.48 | 0.35 | 0.33 |
| N_0_ P_15_ K_15_ | 0.94 | 0.89 | 0.92 | 0.87 | 0.92 | 0.87 |
| N_15_ P_30_ K_15_ | 0.11 | 0.31 | 0.48 | 0.45 | 0.08 | 0.25 |
| N_15_ P_0_ K_15_ | 0.57 | 0.61 | 0.48 | 0.21 | 0.49 | 0.45 |
| N_15_ P_15_ K_30_ | 0.59 | 0.48 | 0.49 | 0.51 | 0.51 | 0.41 |
| N_15_ P_15_ K_0_ | 0.51 | 0.61 | 0.48 | 0.31 | 0.44 | 0.48 |
| N_15_ P_15_ K_15_ | 0.20 | 0.02 | 0.49 | 0.54 | 0.16 | 0.00 |
| N_15_ P_15_ K_15_ | 0.19 | 0.17 | 0.61 | 0.00 | 0.17 | 0.01 |
| N_15_ P_15_ K_15_ | 0.00 | 0.39 | 0.58 | 0.55 | 0.00 | 0.34 |
| N_15_ P_15_ K_15_ | 0.23 | 0.16 | 0.29 | 0.43 | 0.13 | 0.11 |
| N_15_ P_15_ K_15_ | 0.19 | 0.17 | 0.03 | 0.64 | 0.04 | 0.16 |
| N_15_ P_15_ K_15_ | 0.52 | 0.04 | 0.54 | 0.56 | 0.46 | 0.03 |
| N_15_ P_15_ K_15_ | 0.57 | 0.19 | 0.62 | 0.67 | 0.52 | 0.19 |
| N_15_ P_15_ K_15_ | 0.10 | 0.32 | 0.54 | 0.35 | 0.08 | 0.23 |
| N_15_ P_15_ K_15_ | 0.07 | 0.00 | 0.37 | 0.60 | 0.01 | 0.00 |
| N_0_ P_0_ K_0_ | 1.00 | 1.00 | 1.00 | 1.00 | 1.00 | 1.00 |

**Supplementary Table S3.** Transformed values of data in table 2 according to the design (CCRD) of the experiment using formula: $X=1-x$, where $X$ represents transformed value and $x$ represents transformed value of each treatment in table S2.

| **Treatments** | **MDA (nmol mg^-1^ protein)** | | **EL (%)** | | **H_2_O_2_ (nmol mg^-1^ protein)** | |
| --- | --- | --- | --- | --- | --- | --- |
|  | 2016-17 | 2017-18 | 2016-17 | 2017-18 | 2016-17 | 2017-18 |
| N_23.918_ P_23.918_ K_23.918_ | 0.54 | 0.64 | 0.76 | 0.76 | 0.66 | 0.76 |
| N_23.918_ P_23.918_ K_6.082_ | 0.66 | 0.84 | 0.63 | 0.74 | 0.75 | 0.94 |
| N_23.918_ P_6.082_ K_23.918_ | 0.57 | 0.78 | 1.00 | 0.75 | 0.75 | 0.88 |
| N_23.918_ P_6.082_ K_6.082_ | 0.51 | 0.58 | 0.71 | 0.57 | 0.63 | 0.66 |
| N_6.082_ P_23.918_ K_23.918_ | 0.16 | 0.26 | 0.35 | 0.31 | 0.23 | 0.31 |
| N_6.082_ P_23.918_ K_6.082_ | 0.47 | 0.38 | 0.21 | 0.59 | 0.47 | 0.48 |
| N_6.082_ P_6.082_ K_23.918_ | 0.36 | 0.48 | 0.25 | 0.29 | 0.38 | 0.50 |
| N_6.082_ P_6.082_ K_6.082_ | 0.13 | 0.32 | 0.48 | 0.31 | 0.24 | 0.37 |
| N_30_ P_15_ K_15_ | 0.59 | 0.60 | 0.48 | 0.52 | 0.65 | 0.67 |
| N_0_ P_15_ K_15_ | 0.06 | 0.11 | 0.08 | 0.13 | 0.08 | 0.13 |
| N_15_ P_30_ K_15_ | 0.89 | 0.69 | 0.52 | 0.55 | 0.92 | 0.75 |
| N_15_ P_0_ K_15_ | 0.43 | 0.39 | 0.52 | 0.79 | 0.51 | 0.55 |
| N_15_ P_15_ K_30_ | 0.41 | 0.52 | 0.51 | 0.49 | 0.49 | 0.59 |
| N_15_ P_15_ K_0_ | 0.49 | 0.39 | 0.52 | 0.69 | 0.56 | 0.52 |
| N_15_ P_15_ K_15_ | 0.80 | 0.98 | 0.51 | 0.46 | 0.84 | 1.00 |
| N_15_ P_15_ K_15_ | 0.81 | 0.83 | 0.39 | 1.00 | 0.83 | 0.99 |
| N_15_ P_15_ K_15_ | 1.00 | 0.61 | 0.42 | 0.45 | 1.00 | 0.66 |
| N_15_ P_15_ K_15_ | 0.77 | 0.84 | 0.71 | 0.57 | 0.87 | 0.89 |
| N_15_ P_15_ K_15_ | 0.81 | 0.83 | 0.97 | 0.36 | 0.96 | 0.84 |
| N_15_ P_15_ K_15_ | 0.48 | 0.96 | 0.46 | 0.44 | 0.54 | 0.97 |
| N_15_ P_15_ K_15_ | 0.43 | 0.81 | 0.38 | 0.33 | 0.48 | 0.81 |
| N_15_ P_15_ K_15_ | 0.90 | 0.68 | 0.46 | 0.65 | 0.92 | 0.77 |
| N_15_ P_15_ K_15_ | 0.93 | 1.00 | 0.63 | 0.40 | 0.99 | 1.00 |
| N_0_ P_0_ K_0_ | 0.00 | 0.00 | 0.00 | 0.00 | 0.00 | 0.00 |


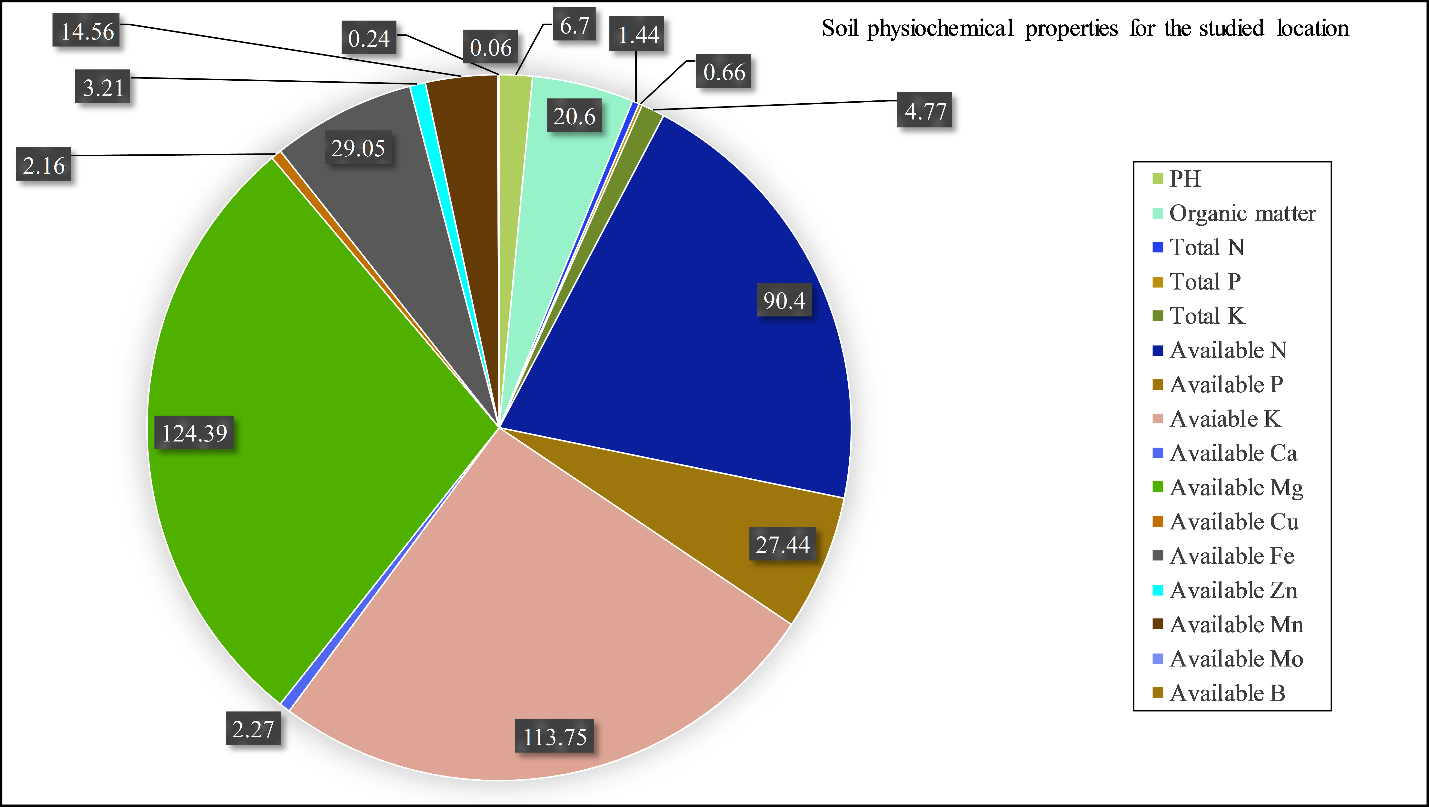

**Supplementary Figure S1.** Soil physicochemical properties for the studied location.
